# Assessment of residual gastric volume using point-of-care ultrasonography in adult patients who underwent elective surgery

**DOI:** 10.1186/s13089-023-00307-8

**Published:** 2023-02-08

**Authors:** T.S. Chaitra, Sanjeev Palta, Richa Saroa, Swati Jindal, Aditi Jain

**Affiliations:** 1 Department of Anaesthesia, Sri Siddhartha Medical College Hospital and Research Centre, Tumkur, India; 2 Department of Anaesthesia, Government Medical College and Hospital, Chandigarh, India

**Keywords:** Gastric ultrasound, Gastric residual volume

## Abstract

**Background:**

Aspiration pneumonitis remains a dreaded complication that may lead to almost 9% of anaesthesia-related deaths. The presence of gastric contents has always been a contributing factor to an increased risk of aspiration. Preoperative gastric ultrasound has been suggested as a modality for determining residual volume in special populations and conditions. We conducted an observational study to determine the gastric residual volume in preoperative patients of elective surgery with gastric ultrasound and to study its correlation with patient factors.

**Methods:**

We enrolled 411 patients in the age group of 18–80 with ASA-PS I and II having BMI less than 35 kg/m^2^. Patients with prior gastrointestinal surgery and parturients were excluded from the present study. Gastric antrum in both supine and right lateral decubitus positions was measured using USG in the immediate preoperative period, and gastric residual volume was calculated, which was subsequently correlated with various patient factors.

**Results:**

On qualitative assessment, 97 and 118 patients were observed to have distended stomachs in the supine and right lateral decubitus positions, respectively. On quantitative assessment, 336 had safe GRV, 60 patients were classified as having a low risk of aspiration (GRV < 1.5 ml/kg) while 13 had a high risk of aspiration (> 1.5 ml/kg). Eight patients with a fasting duration of more than ten hours and five who fasted between 6 and 10 h had a gastric residual volume of more than 1.5 ml/h. Patients who were premedicated with histamine blockers had a statistically significant higher antral cross-sectional area (*p*-value − 0.022*) and GRV (*p*-value − 0.018*) in the right lateral decubitus position compared to patients who had taken proton pump inhibitors (PPIs). As BMI increased, there was a statistically significant (*p*-value < 0.001) increase in mean antral CSA in both supine and right lateral decubitus positions. There was a statistically significant association found between type 2 diabetes (*p*-value 0.045*) with antral grade.

**Discussion:**

Patients can have significant residual volume (> 1.5 ml/kg) despite adequate fasting, and preoperative gastric ultrasound can help in assessing the same and guiding perioperative airway management. PPIs are more effective in reducing gastric residual volume as compared to histamine blockers. Patients with a BMI of more than 30 and type 2 diabetes mellitus have significant correlation with increased gastric residual volume mandating preoperative gastric ultrasound assessment for effective management.

**Conclusions:**

Patients with BMI over 30 and type 2 diabetes may benefit from POCGUS to guide perioperative airway management by stratifying GRV.

*Trial registration* Name of registry-Clinical Trial Registry of India. Trial registration number-2020/03/024083. Date of registration-19.3.2020. URL-http://ctri.nic.in/Clinicaltrials/pmaindet2.php?trialid=39961&EncHid=&userName=

## Introduction

Aspiration pneumonitis is an anaesthetic catastrophe with mortality ranging up to 5% and accounts for almost 9% of all anaesthesia-related deaths [1, 2]. Nearly 50% of airway-related deaths in anaesthesia are directly due to the aspiration of gastric contents secondary to the development of significant pulmonary complications. It occurs more frequently in patients with underlying risk factors during induction of anaesthesia or airway instrumentation [1]. The presence of gastric contents increases the risk of aspiration. As a result, practice guidelines for preoperative fasting were developed to provide adequate time for gastric emptying in patients undergoing surgery. The practice guidelines of the American Society of Anesthesiologists (ASA) for healthy adults consider a minimum fasting duration of 2 h (h) for clear fluids, 6 h for a light meal, and 8 h for a fatty meal, fried foods or meat for the safe conduct of anaesthesia [3]. However, despite following these guidelines, the incidence of pulmonary aspiration is 1:4000 in healthy, fasted individuals [4].

Many techniques have been described to assess gastric contents, like nasogastric tube aspiration of gastric contents, paracetamol absorption, and electrical impedance tomography [5]. Because of cost and radiation exposure, scintigraphy has remained restricted mainly to research purposes. The other methods, except for the Ryles tube aspiration technique, are not suitable for the perioperative period owing to their complexity. Point-of-care gastric ultrasound preoperative gastric ultrasound represents a feasible option that can be incorporated into standard anaesthesia practice to assess the gastric antrum and, thereby, gastric volume owing to its cost-effectiveness and ease of performing at the bedside. It can thus help the anaesthetist to anticipate the aspiration risk and guide anaesthetic and airway management more appropriately.

Currently, airway management of some groups of patients, like the critically ill, patients with diabetic gastroparesis, patients with neuromuscular disorders and those with advanced liver or renal dysfunction, is mainly based on the assumption of a full stomach [6]. Preoperative gastric ultrasound may be helpful in these high-risk patients and special populations in whom nil per oral (NPO) status is difficult to confirm [7, 8]. Although preoperative gastric ultrasound cannot replace strict adherence to current fasting guidelines, it can be a valuable tool for clinical decision-making whenever there is uncertainty about the gastric contents [6]. The use of preoperative gastric ultrasound as a routine for preoperative evaluation of residual volume is still controversial and there is a lacunae in the current clinical data for its use in elective surgical patients [4].

Therefore, we planned this study to assess the gastric residual volume (GRV) preoperatively in patients posted for elective surgery to investigate those at high risk for aspiration and to correlate various patient factors with this increase in risk.

## Methodology

The present study was conducted to assess the GRV of adequately fasted patients scheduled for elective surgery and their correlation with various preoperative factors in a tertiary care hospital in North India. After the study obtained approval from the Institutional Review Board, it was prospectively registered in the Clinical trial registry of India (CTRI/2020/03/024083).

### Study population

We enrolled 411 patients scheduled to undergo elective surgery who were more than 18 years of age with ASA Physical Status I or II. Exclusion criteria include patients with a history of esophageal or gastric surgery, those with abnormalities of the gastrointestinal tract such as hiatal hernia, gastric tumours, parturients, and those with a body mass index (BMI) > 35 kg/m^2^.

### Conduct of the study

Patients fulfilling the inclusion and exclusion criteria were identified in the ward a day before the surgery. The procedure of gastric ultrasound examination was explained, and written informed consent was taken. Patients were asked to maintain fasting for 8 h for solid meals and 2 h for clear liquid, as is the standard institutional protocol. On the day of surgery in the preoperative area, the hours of fasting and the premedication history were noted.

Preoperative gastric ultrasound was performed in the preoperative area of the operation theatre complex in all the patients enrolled for the present trial. The observer performed all the scans under the supervised guidance of an experienced anaesthesiologist who was well versed in ultrasound. All observations were noted by the observer with confirmations from the supervisor. A low-frequency (2–5 MHz) curvilinear array probe (SonoSite, Inc. Bothell, WA 98,021 USA/ Esaote Asia `pacific, Mylab one, 91,125,082) was employed to scan the gastric area using abdominal scan mode settings.

**Fig. 1:**
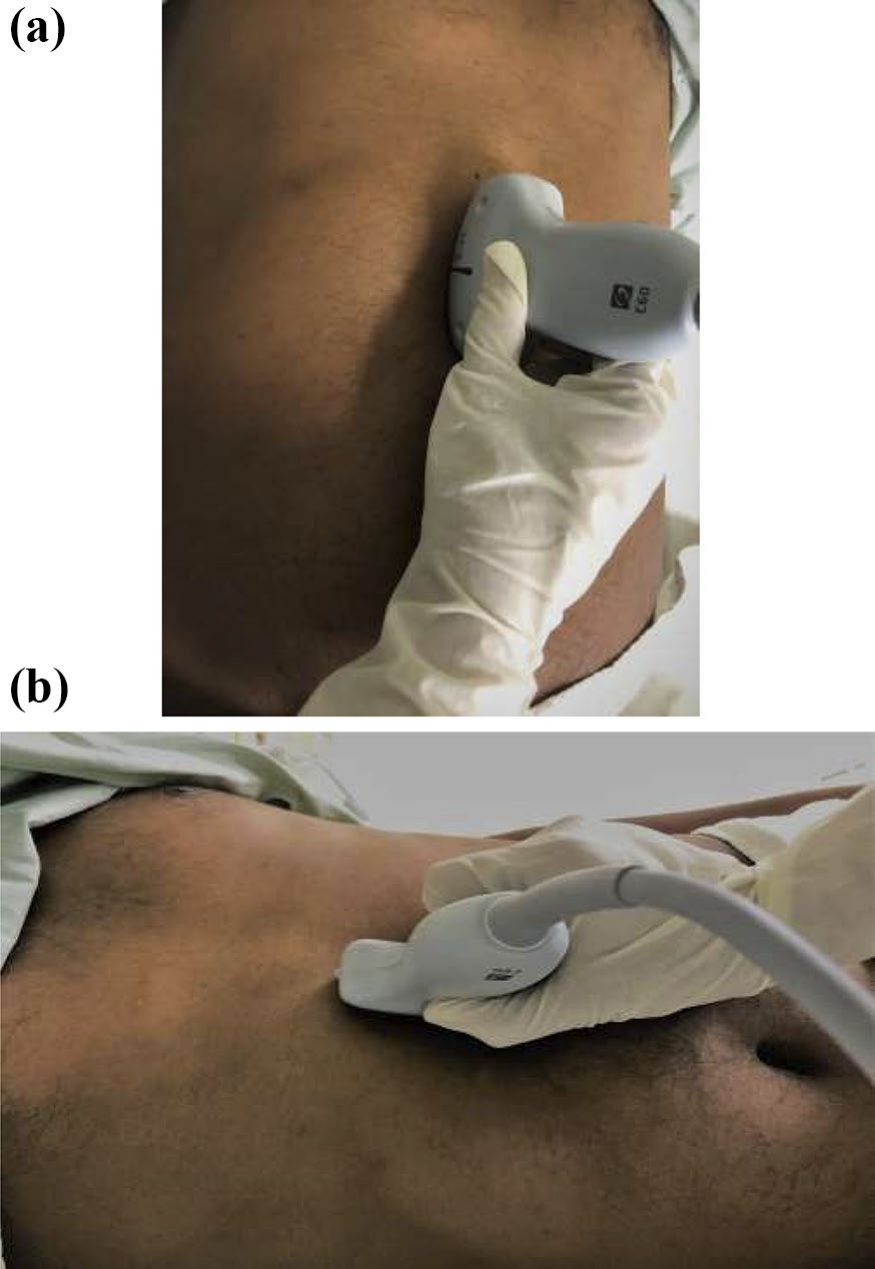
**a** Scanning in supine position. **b** Scanning in right lateral decubitus position

The epigastrium was scanned in a sagittal plane sweeping the transducer from the left to right subcostal margins to locate the gastric antrum. Scanning was first done in the supine position, followed by scanning in the right lateral decubitus (RLD) position (Fig. 1). The gastric antrum was identified just below the left lobe of the liver and pancreas posteriorly, where the aorta/superior mesenteric artery/inferior vena cava act as an important landmark. Care was taken to avoid oblique views from transducer over-rotation that could overestimate antrum size. This protocol was adapted from the one described by Haskins et al. [6].

**Fig. 2:**
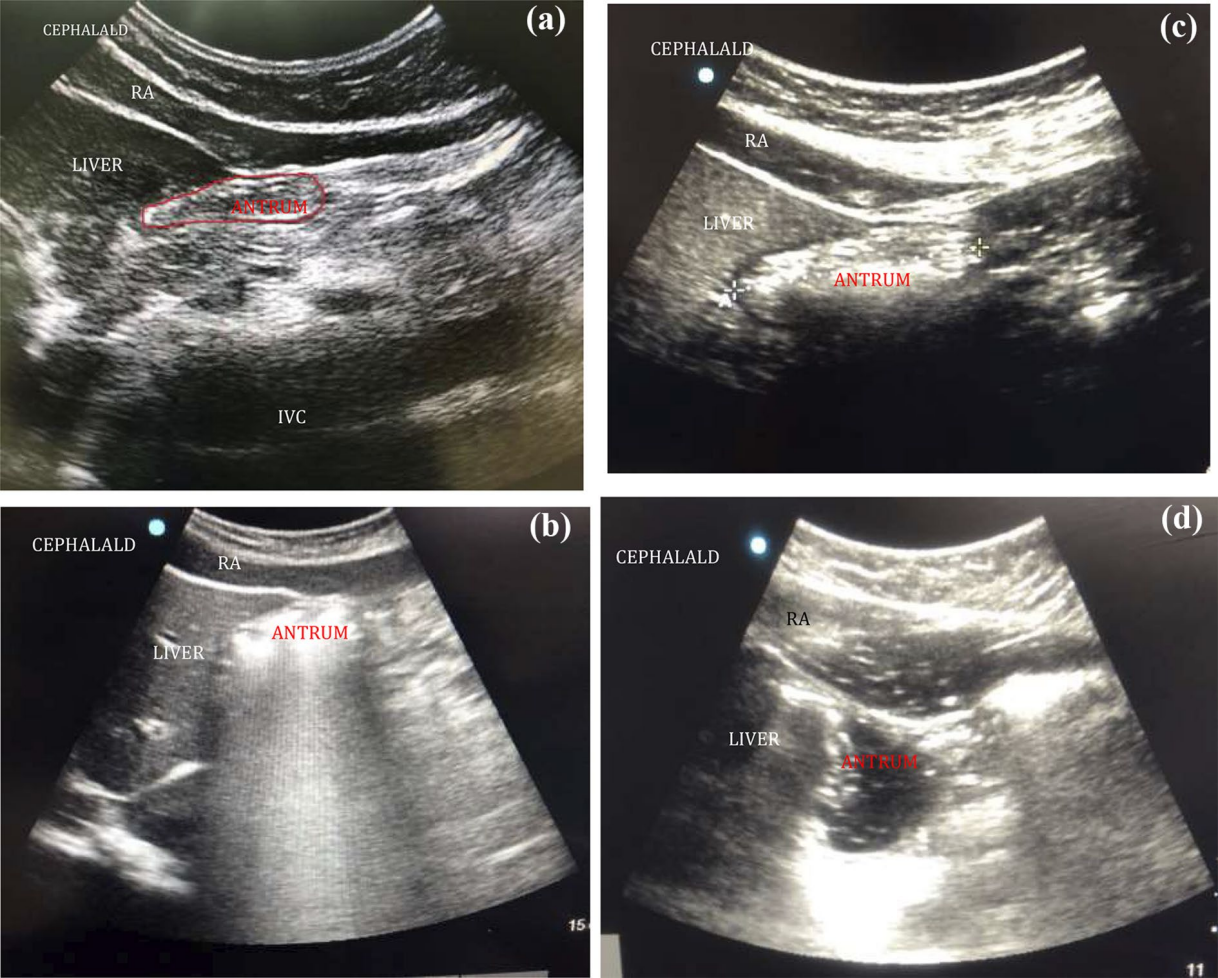
**a** Sonoanatomy of empty gastric antrum. Antrum appears flat or bull’s eye shaped structure; RA (rectus abdominis), IVC (inferior vena cava). **b** Sonoanatomy of distended antrum. In early-stage, solids resembles ground glass appearance, the posterior wall of antrum is obscured; RA (rectus abdominis). **c** Sonoanatomy of distended antrum. In late-stage, solids appear heterogenous and hyperechoic (rectus abdominis). **d** Distended antrum with clear fluids and gas having/hypoechoic appearance (“starry night”)

**Fig. 3:**
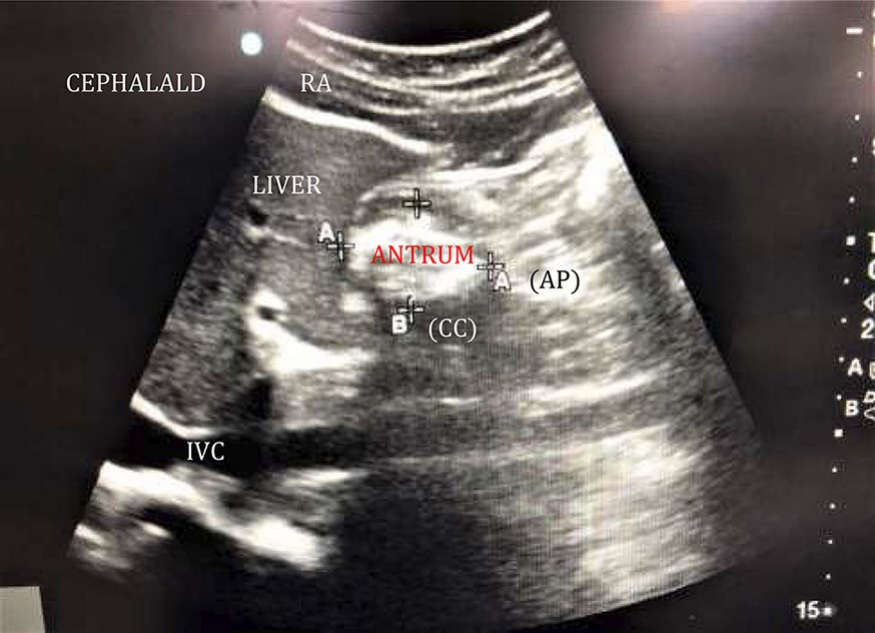
Two diameter method—Anteroposterior (AP) and Craniocaudal (CC) measurements of antrum; RA (rectus abdominus), IVC (inferior venacava)

According to the appearance on the USG, the contents were identified as either empty, clear liquids or solids (Fig. 2). If the walls of the antrum were found opposing each other or giving a bull's eye/target pattern, it was considered empty. If the antrum was not empty, a qualitative assessment of the contents was done. The clear fluids (such as normal gastric secretions, tea, or water) gave an anechoic (black) appearance on USG. Liquids with multiple gas bubbles had “starry night” appearance. In the early stage, solid/particulate matter gave—a “frosted glass” pattern with multiple ring-down artefacts obscuring the posterior antrum. In the later stage, solid contents appeared more heterogenous, particulate, and hyperechoic. All images were obtained between peristaltic contractions of the antrum. After qualitative analysis of the antrum, cross-sectional antral area (CSA) was calculated. The measurements were made at the aorta or inferior vena cava level using the USG machine in supine and RLD positions. The traditional two-diameter method was used, which involved measuring two perpendicular diameters of the antrum, from serosa to serosa, longitudinal or craniocaudal (CC), and the anteroposterior(AP) using the formula developed by Bolondi et al. [9] in which (Fig. 3).

$${\text{Antral CSA}}\, = \,\left( {{\text{CC }} \times {\text{ AP}}} \right) \, \times \, \pi )/{4},\quad {\text{with }}\pi {\text{ value}}\, = \,{3}.{14}.$$

After performing preoperative gastric ultrasound, the quantitative GRV was calculated for each patient using the formula by Perlaset al. [10]. Stomach volume (ml) = 27 + 14.6 ACSA (cm^2^) − 1.28 age (years) (ACSA—antral cross-sectional area). The risk of aspiration was assessed qualitatively by the antral grading system [11].

### Statistical analysis

The sample size was calculated using the formula *N* = (*Z* × SD/*E*)^2^, where *N* = number of patients, *Z* = standard normal deviate, 1.96; SD = standard deviation of gastric content volume (48.31 ml), *E* = desired margin of error (5%). Taking 48.31 ml from a previous study as the standard deviation of gastric content volume and 5% as margin of error, the sample size was calculated to be 358 [9]. Assuming a 15% drop-out rate, we decided to enrol 410 patients in our study. The data were entered in the MS EXCEL spreadsheet, and analysis was conducted using IBM SPSS STATISTICS (version 22.0). The normality of the variables (quantitative data) was tested with the Shapiro–Wilk test/Kolmogorov–Smirnov test. The quantitative variables were compared using the Mann–Whitney test for two groups and Kruskal–Wallis tests for more than two groups. Categorical variables were reported as counts and percentages. Spearman correlation coefficient was calculated to see the relation of quantitative variables of 2 sides. The qualitative variables were compared using the Chi-square or Fisher’s exact test. All the statistical tests were two-sided and were performed at a significance level of 5%. A *p*-value < 0.05 was considered significant.

## Results

**Fig. 4:**
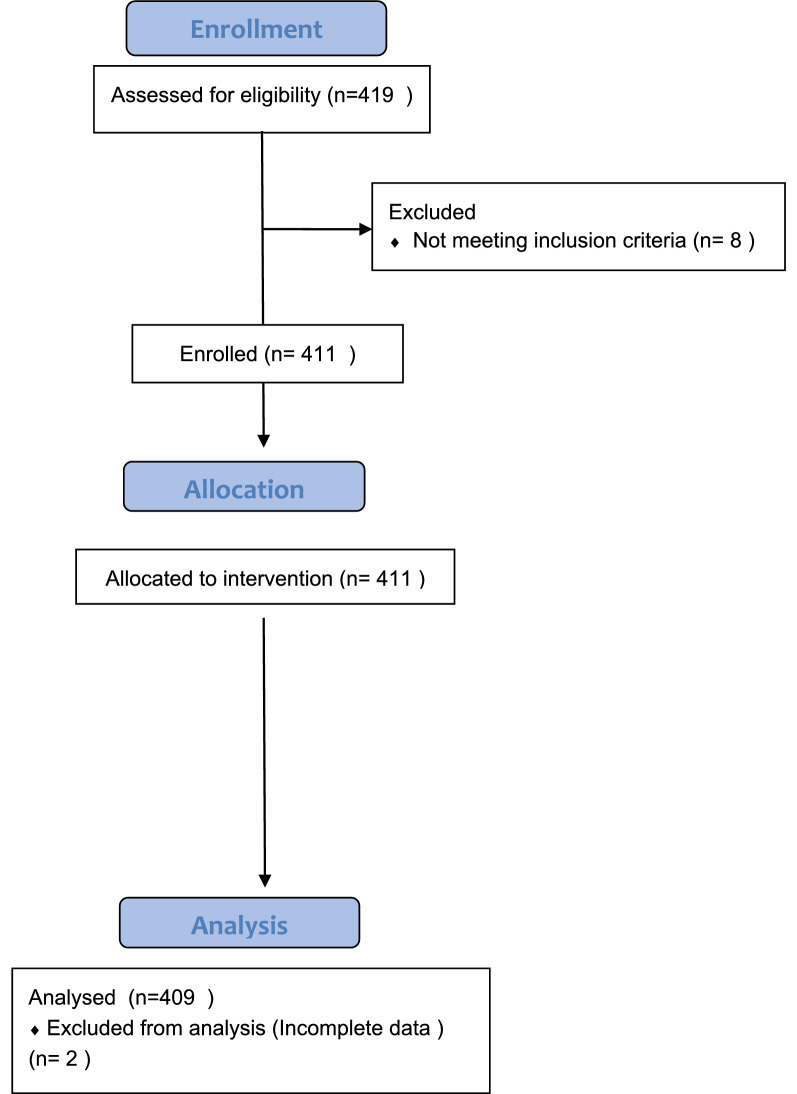
CONSORT flow diagram

We screened 419 patients in the age group of 18–80 years of ASA physical status I and II scheduled to undergo elective surgery under general or regional anaesthesia. 411 were included for assessing the GRV using gastric ultrasound as they fulfilled the inclusion criterion. Two patients were excluded from the analysis due to incomplete data (Fig. 4).

### Characteristics of the subjects

The mean age of our study population was 42.2 years. There were more males (55%) than females in our study population. Although we did not recruit patients with a BMI of more than 35%, more than half the population had a body mass index (BMI) of more than 25 and was classified as overweight (BMI > 25 and < 30) or obese (BMI 30–35). 8.1% of the population had a BMI of under 18 and was classified as underweight. 43.7% of the patients suffered from comorbidities such as hypertension, diabetes mellitus type 2, hypothyroidism and obesity. The mean fasting time of patients for solid food was 11 h, and it was 2 h for clear fluids. All patients received premedication for aspiration prophylaxis the night before surgery and the morning of surgery. Most patients (91.7%) received proton pump inhibitors (PPI), and 8.3% received histamine blockers (Table 1).

**Table 1:** Characteristics of study population

Characteristics	
Age (years)	43.23 ± 14.24
Fasting time (h)	Mean ± SD
Solid	11.09 ± 1.52
Liquid	2.08 ± 0.45
Gender	*N* (%)
Male	225 (55)
Female	184 (45)
BMI (kg/m^2^)	*N* (%)
Under weight (< 18.5)	33 (8.1)
Normal weight (18.5–24.9)	168 (41.1)
Over weight (25–29.9)	153 (37.4)
Obese (30–35)	55 (13.4)
ASA-PS	*N* (%)
ASA-PS I	212 (51.8)
ASA-PS II	197 (48.2)
Premedication	*N* (%)
H2 blocker	34 (8.3)
Proton pump inhibitors	375 (91.7)

### Gastric ultrasound observations

Preoperative gastric ultrasound was performed in both supine and RLD positions in the sagittal plane. A qualitative assessment was performed, including the content type and antral shape. This was followed by a quantitative evaluation which involved antral measurements in both anteroposterior and craniocaudal diameters, which were used to calculate antral CSA, and then the estimated GRV was derived.

#### 1. Qualitative assessment

Preoperative gastric ultrasound qualitative assessment involved identifying different antral shapes and describing the content based on echogenicity. Flat and bull’s eye appearance of antrum was considered empty and distended appearance was viewed as the presence of contents which were further described as solid (hyperechoic), liquid (hypoechoic) or mixed (hyperechoic and hypoechoic). 97 and 118 patients were found to have distended stomachs in the supine and right lateral position, respectively (Table 2).

**Table 2:** Antral shape in supine and RLD position

Shape	Supine position		RLD position	
	N	%	N	%
Flat	152	37.2	145	35.5
Bulls eye	160	39.1	148	36.2
Distended	97	23.7	116	28.4

#### 2. Quantitative assessment

The antral measurements and the calculated gastric residual volume are given in Table 3.

**Table 3:** Quantitative measurements in supine and RLD position

Measurements (mean ± SD)	Supine	RLD
Anteroposterior diameter in cm	3.01 ± 0.76	3.10 ± 0.80
Craniocaudal diameter in cm	1.94 ± 0.68	2.02 ± 0.70
Antral CSA in cm^2^	4.52 ± 2.24	4.81 ± 2.03
Gastric residual volume	36.46 ± 25	42.13 ± 29.06

On calculating the aspiration risk, we found 336 patients had a safe gastric residual volume, and 60 patients had a gastric volume of less than 1.5 ml/kg, which implies a low risk for aspiration. However, 13 patients had a GRV of more than 1.5 ml/kg, making them more prone to aspiration.

#### 3. Correlation of factors with gastric ultrasound

We divided our study population into those whose fasting hours were less than 10 h and those in whom it was more. On comparing their gastric volumes, the difference was not found to be statistically significant. Eight patients with a fasting duration of more than 10 h and five patients who fasted between 6 and 10 h had a GRV of more than 1.5 ml/h, thus potentially making them high-risk candidates for pulmonary aspiration.

We found that patients premedicated with histamine blockers had a statistically significant higher antral CSA (*p*-value-0.022*) and GRV (*p*-value − 0.018*) in the RLD position (antral CSA − 5.49 ± 2.14; GRV − 51.84 ± 35.49) as compared to those who had taken PPIs (antral CSA − 4.75 ± 2.02; GRV − 41.25 ± 28.30).

**Fig. 5:**
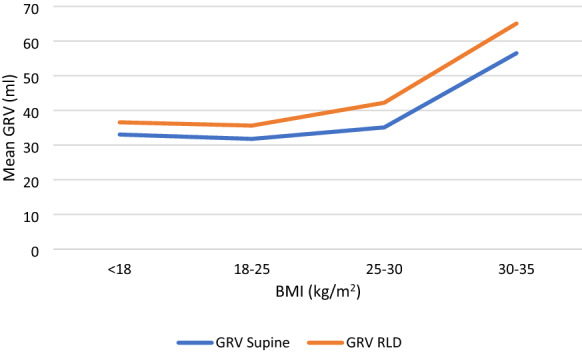
Correlation of GRV with BMI

Patients were divided into four groups according to their BMI. They included, underweight (N-33; BMI < 18.5 kg/m^2^), normal weight (N-168; BMI 18.5–25 kg/m^2^), overweight (N-153; BMI 25–30 kg/m^2^) and obese (N-55; BMI 30–35 kg/m^2^). In overweight patients, the mean antral CSA in supine and RLD positions were 4.50 ± 1.57 and 4.94 ± 1.96, respectively. In obese patients, the mean antral CSA in supine and RLD positions were 6.04 ± 2.22 and 6.59 ± 2.30, respectively. As BMI increased, there was a statistically (*p*-value < 0.001**) significant increase in mean antral CSA in both supine and RLD positions. The mean GRV in supine and RLD positions in overweight patients were also found to be 35.11 ± 23.60 and 42.22 ± 31.39, respectively. In obese patients, the mean GRV in supine and RLD positions was 56.51 ± 30.06 and 65.07 ± 31.03, respectively. As BMI increased, there was a statistically (*p*-value < 0.001**) significant increase in mean GRV in both supine and RLD positions (Fig. 5).

After a qualitative and quantitative assessment of the stomach was made using ultrasound, patients were classified according to the antral grading system using estimated GRV. Different patient factors, including age, sex, BMI, ASA physical status, fasting duration for solids, fasting duration for liquids and type 2 diabetes mellitus, were subjected to univariate analysis to identify any influence of these factors on antral grade. It was observed that there were no statistically significant association between age (*p*-value − 0.74), sex (*p*-value − 0.99), ASA physical status (*p*-value − 0.13), fasting hours for solids (*p*-value − 0.18) and liquids (*p*-value − 0.15) with antral grade. However, there was a statistically significant association found between BMI (*p*-value − 0.026*) and type 2 diabetes (*p*-value 0.045*) with antral grade. Among 409 patients, 32 were type 2 diabetic, and 3/32 (9.37%) were found to have GRV > 1.5 ml/kg. Five overweight and one obese patient also had a GRV > 1.5 ml/kg (Table 4).

**Table 4:** Univariate analysis of antral grade with patient factors

	Antral grade			*p*-value
	Empty—safe	Low risk	High risk	
Number of patients (%)	336 (82.2)	60 (14.70)	13 (3.2)	
Age (years) mean ± SD	43.39 ± 14.09	41.97 ± 14.70	45.08 ± 16.69	0.74
Sex (M:F) N	185:151	33:27	7:6	0.99
BMI (kg/m^2^) N				
< 18.5	26	4	3	0.026*
18.5–24.9	47	17	4	
25–29.9	124	24	5	
30–35	39	15	1	
ASA (I: II) N	181: 155	27: 33	4: 9	0.13
Fasting time for solids (h), mean ± SD	11.04 ± 1.52	11.40 ± 1.48	10.96 ± 1.49	0.18
Fasting time for liquids (h), mean ± SD	2.07 ± 0.42	2.13 ± 0.59	2.15 ± 0.37	0.15
T2DM (N)	22	7	3	0.045*

*p < 0.05 i.e. statistically significant

## Discussion

Preoperative gastric ultrasound has recently been suggested to determine gastric volume in perioperative conditions for situations where prandial status is uncertain, like cognitive dysfunction, language barriers, and paediatric patients.

In the present study, the mean GRV in supine and RLD positions was found to be 36.46 ± 25 and 42.13 ± 29.06, respectively. It was observed that 82.2% patients (*N* = 336) had grade 0 antrum (empty or safe GRV) before scheduled surgery, 14.7% patients (*N* = 60) had a grade 1 antrum (GRV < 1.5 ml/kg) and 3.2% patients (*N* = 13) had grade 2 antrum with GRV of > 1.5 ml/kg. Our findings are similar to Ohashi et al. [12]. They studied 203 patients posted for elective surgery with a fasting period of 6 h for solids and 2 h for clear fluids. They found that 91.4% of patients (*N* = 203) had grade 0 antrum before the scheduled surgery, 5.9% of patients (*N* = 13) had a grade 1 antrum, and 2.7% of patients (*N* = 6) had grade 2 antrum with GRV of > 1.5 ml/kg [12]. We enrolled almost double the number of patients in their study for more robust evidence.

When we compared the GRV with respect to fasting times, we found that patients who had fasted between 10 and 15 h had greater GRV (42.80 ± 28.83) than those who had fasted between 6 to 10 h (40.78 ± 29.59). Even though there was no statistically significant association between GRV and antral grade with fasting times (*p*-value − 0.203), 13 patients had GRV > 1.5 ml/kg. Five of the 13 patients had fasted according to fasting guidelines, and eight had fasted for more than 10 h. While this finding initially seems counterintuitive, the authors rationalise that this may be due to the continuous secretions of ghrelin that stimulates gastric juices, which in turn increase when the patient is hungry. This implies that prolonged fasting of more than the recommended 8 h may also increase the gastric residual volume, and adherence to fasting guidelines does not always ensure an empty stomach. While our institute tries to follow a liberal fasting regime, our multi-speciality hospital caters to surgeries from various surgical specialities with long surgical lists. Predicting the timing of an operation is complex as the surgical schedule is subject to frequent changes because of emergencies or cancellations.

Our study found that patients premedicated with ranitidine compared to PPI had a higher GRV. The authors hypothesise that this may be because PPIs act on terminal receptors and have a more direct action than histamine blockers. The number of patients premedicated with ranitidine is relatively low compared to PPIs. This drug is now avoided in our routine clinical practice after the FDA warning regarding NDMA contamination. In a meta-analysis by Clark et al., the authors found ranitidine to be more efficacious in decreasing GRV than PPI. They, however, did not state any clinical implication for the same and mentioned that further research is needed [13].

Our study observed that as BMI increased, there was a statistically significant increase in mean antral CSA in both supine and RLD positions (*p*-value < 0.001) In their study, Sharma et al. also found that as the BMI increased from 25 to 35 kg/m^2^, there was a steady rise in antral CSA and GRV in both supine and RLD positions. A significant increase was found only in supine antral CSA in their study (*p*-value − 0.026*) [11]. However, in a study by Mohammed et al., a significant difference in GRV (*p*-value − 0.0001**) was found only in the RLD position when they compared patients with BMI > 30 to those less than 30 [14].^.^ This variation in their study may be due to the involvement of more patients with ASA III physical status, BMI > 35 kg/m^2^, and not premedicating the patients before surgery in their study. A study was done by Sadhvi et al. which included 33 patients with a BMI > 30 kg/m^2^ and five patients with a BMI > 35 kg/m^2^, amongst which 27.8% of obese patients and 11.4% of nonobese patients were at risk of aspiration (*p* < 0.007**) with mean GRV 1.9 ± 0.52 ml/kg. The authors found that a multivariate regression analysis between BMI and gastric contents showed a robust linear association (*p* = 0.0003**) and also that this association was even stronger in the obese patient subgroup (*p* < 0.0001**) [15]. Our findings are consistent with this study. Based on our current results, we suggest performing the POGUS in both supine and right lateral decubitus patients for a patient with an increased BMI of more than 30.

Per univariate analysis, we found a statistically significant association between BMI (*p*-value − 0.026*) and type 2 diabetes (*p*-value − 0.045*) with antral grade. Our findings are consistent with the study conducted by Zhou et al., which was a prospective cohort study in 2017 where they included 52 type 2 diabetic and 50 non-diabetic patients posted for elective surgeries [16]. Patients in both groups were fasted overnight (at least 10 h) from the last meal. Preoperative gastric ultrasound was performed 2 h after ingesting clear fluid or 6 h after a light meal. Authors found that type 2 diabetic patients (25/52; 48.1%) have a higher prevalence (*p* = 0.000) of the full stomach when compared to non-diabetic patients (4/50; 8.00%) [16]. Bouvier et al. conducted a study involving 440 patients undergoing elective or emergency surgery. Univariate analysis of patient factors showed a significant statistical association between obesity (*p*-value − 0.001**), and diabetes mellitus (*p*-value − 0.012*) [17]. Although gastroparesis in diabetic patients is known, its associations and pathogenesis are still under research. Few of the mechanisms that have been postulated are autonomic neuropathy leading to gastric motor dysfunction; enteric neuropathy; abnormalities of the interstitial cells of Cajal and the use of incretin-based medication [18].

Our study has a few limitations. Since ultrasonography is a subjective interpretation, it can only be used as an adjunct to making clinical decisions. Although we used a single researcher in the presence of another experienced anaesthesiologist to eliminate observer bias, we did not get the all the images evaluated by a second person. Our primary observer had received training for 6 months prior to the initiation of this study and was supervised by another with experience of more than 10 years in anaesthesiology and ultrasonography. We did this to promote a more practical method, as in routine clinical practice, the treating anesthesiologist will generally make a clinical judgement based on their findings. Our observations were made only in ASA I–II patients; hence generalisation of results is not possible concerning ASA III and IV patients. Our study population was relatively young (mean age − 42.2 years) so extrapolation of our findings to patients posted for surgery at extremes of age might require further study. We did not compare ultrasonography with any other method for estimating residual volume.

Ours is one of the largest study, including more than 400 subjects, done for evaluation of gastric volume in preoperative patients. We found that despite adherence to fasting guidelines and aspiration prophylaxis premedication, some patients still have significant gastric residual volume. This could lead to a change in airway management strategy, such as adopting a rapid sequence induction and intubation (RSII) or shifting from sedation with an unprotected airway to that with a definitive airway.preoperative gastric ultrasound is a simple, cheap, and convenient radiographical tool which can help perioperative physicians identify those patients that warrant a change in the anaesthesia technique. While the authors realise that for formal recommendations much larger population-based studies are needed, we believe that a preoperative scan, especially in patients with higher BMI, diabetes mellitus and possibly prolonged fasting times helps in identification of patients who are at a higher risk for aspiration and may lead to a change in the perioperative plan for a safer outcome.

## Data Availability

Data available on request.

## Abbreviations

ASA: American Society of Anesthesiologists
POCGUS: Point-of-care gastric ultrasound
NPO: Nil per oral
GRV: Gastric residual volume
RLD: Right lateral decubitus
CSA: Cross-sectional area
CC: Craniocaudal
AP: Anterio-posterior
BMI: Body mass index
PPI: Proton pump inhibitors
